# Supplementary material for: Genome-wide genetic and epigenetic analyses of pancreatic acinar cell carcinomas reveal aberrations in genome stability
Source: Nat Commun. 2017 Nov 6;8:1323. doi: 10.1038/s41467-017-01118-x (PMC5673892; doi:10.1038/s41467-017-01118-x)
Supplement: Supplementary file 1 — Supplementary Information [file 41467_2017_1118_MOESM1_ESM.pdf]

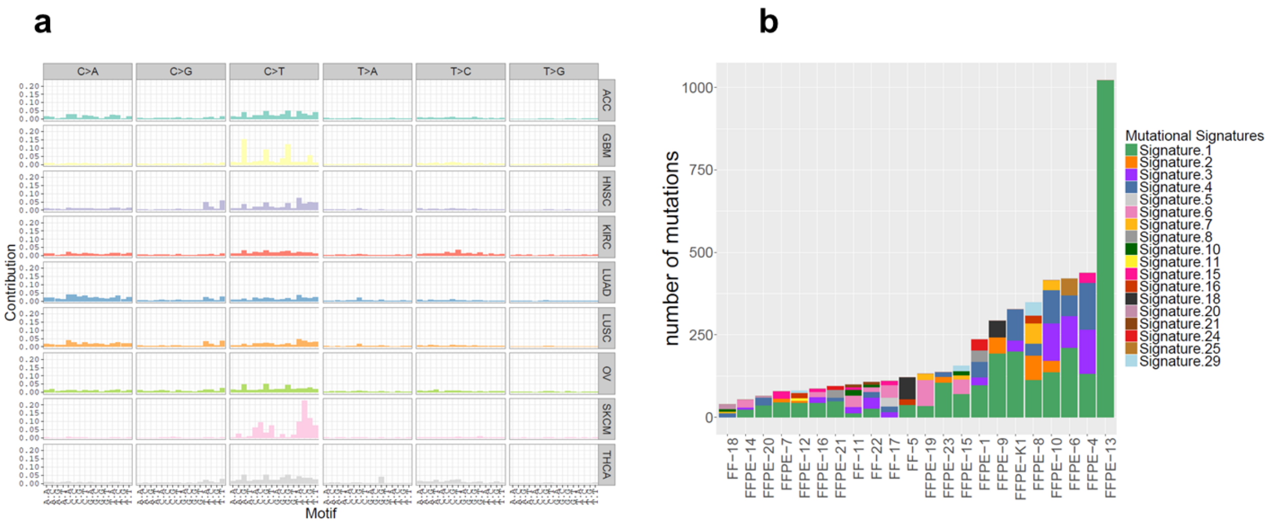

Supplementary Figure 1

Whole exome sequencing shows mutational signatures in ACC are associated with smoking, defects in double-strand breaks and deamination of 5-methylcytosine.

a. Frequency plot of point mutations in the context of one base pair up- and one base pair downstream in ACC compared to data from other cancer entities generated by TCGA. GBM: Glioblastoma Multiforme, HNSC: Head and Neck Squamous Cell Carcinoma, KIRC: Kidney Renal Clear Cell Carcinoma, LUAD: Lung Adenocarcinoma, LUSC: Lung Squamous Cell Carcinoma, OV: Ovarian serous cystadenocarcinoma, SKCM: Skin Cutaneous Melanoma, THCA: Thyroid carcinoma. **b.** Published mutational signatures occurring in each sample is displayed and sorted by numbers of mutations per tumor.

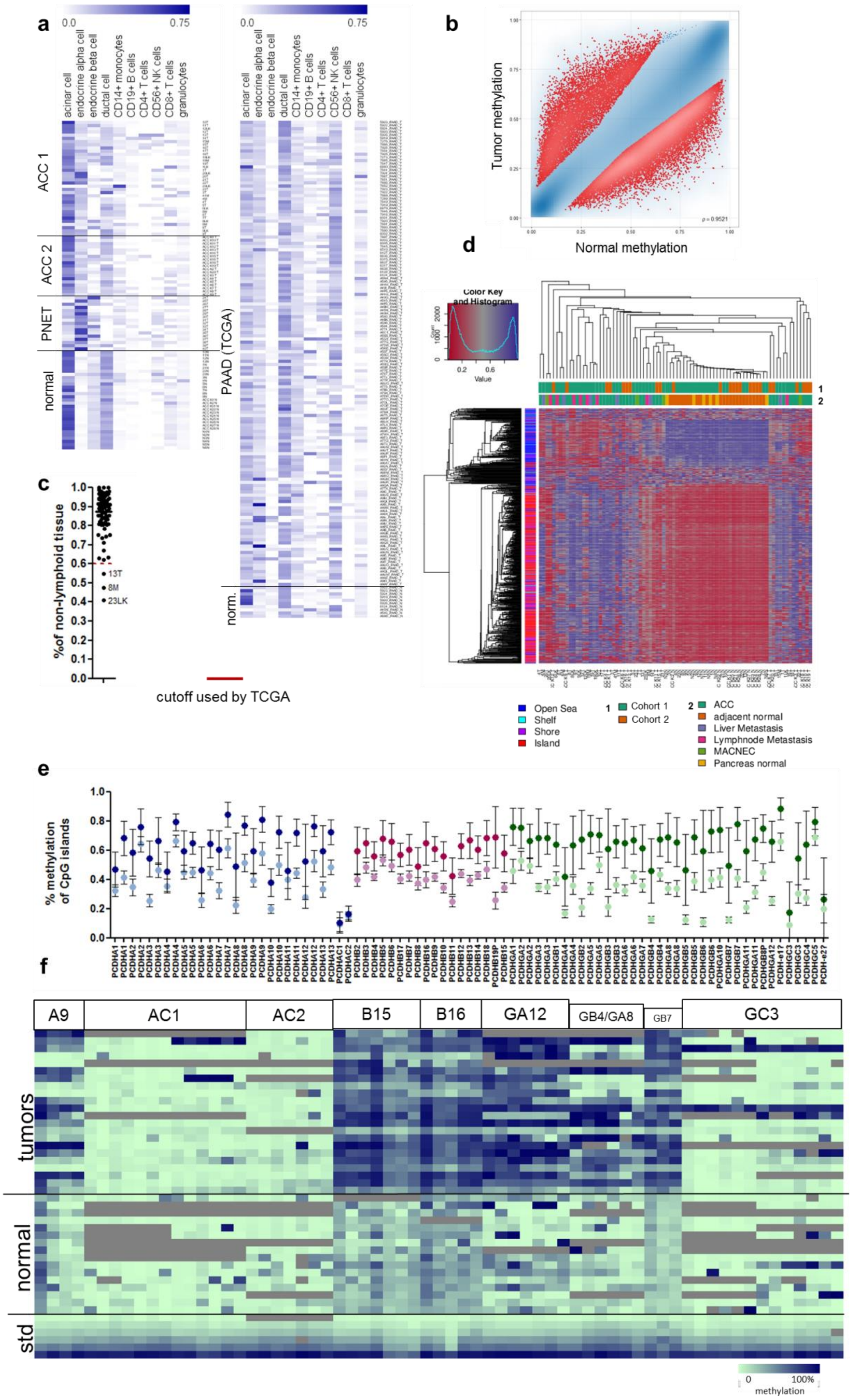
a
0.0
0.75
acinar cell
endocrine alpha cell
endocrine beta cell
ductal cell
CD14+ monocytes
CD19+ B cells
CD4+ T cells
CD56+ NK cells
CD8+ T cells
granulocytes
ACC 1
ACC 2
PNET
normal
PAAD (TCGA)
norm.
b
Tumor methylation
Normal methylation
c
%of non-lymphoid tissue
13T
8M
23LK
cutoff used by TCGA
d
Color Key and Histogram
Count
Value
1
2
Open Sea
Shelf
Shore
Island
Cohort 1
Cohort 2
ACC
adjacent normal
Liver Metastasis
Lymphnode Metastasis
MACNEC
Pancreas normal
e
% methylation of CpG islands
f
A9
AC1
AC2
B15
B16
GA12
GB4/GA8
GB7
GC3
tumors
normal
std
0
100%
methylation

Supplementary Figure 2
Cell type contributions, global DNA methylation and quality of ACC.
a. cell type contributions of sorted pancreatic cells (acinar, duct, α- and β-cells) and blood cells (monocytes, B cells, CD4+ and CD8+ T cells, natural killer (NK) cells and granulocytes) in ACC, PNET, PDAC and normal pancreatic tissue based on methylation profiles. Scale refers to the percentage of contribution of each sorted cell type to the tumors normalized to 1. **b.** Methylation scatterplot of all CpG sites on the 450K array in ACC cohort 2 reveal massive differential methylation comparing all primary tumors versus all normal tissues. (Red: sites are above the rank cutoff, e.g. are considered biologically significant, blue: transparency corresponds to density of sites). **c.** Tumor purity estimate measured by LUMP (for details refer to supplementary methods). **d.** Correlation-based clustering of all single CpG-sites (agglomeration strategy: average) with annotations of cohort (lane 1) and tissue type (lane 2). MACNEC: mixed acinar-neuroendocrine carcinoma. **e.** 700 kb hypermethylation of the CpG islands of the PCDH cluster measured by the 450K array. Each PCDH member harbors two CpG islands. Blue: PCDH-α, red: PCDH-β, green: PCDH-γ. Light colors represent the average of normal tissue, dark colors tumor tissues. Error bars: +/- standard error of the mean. **f**. hypermethylation of members of the PCDH cluster measured by MassARRAY. Grey: missing values.

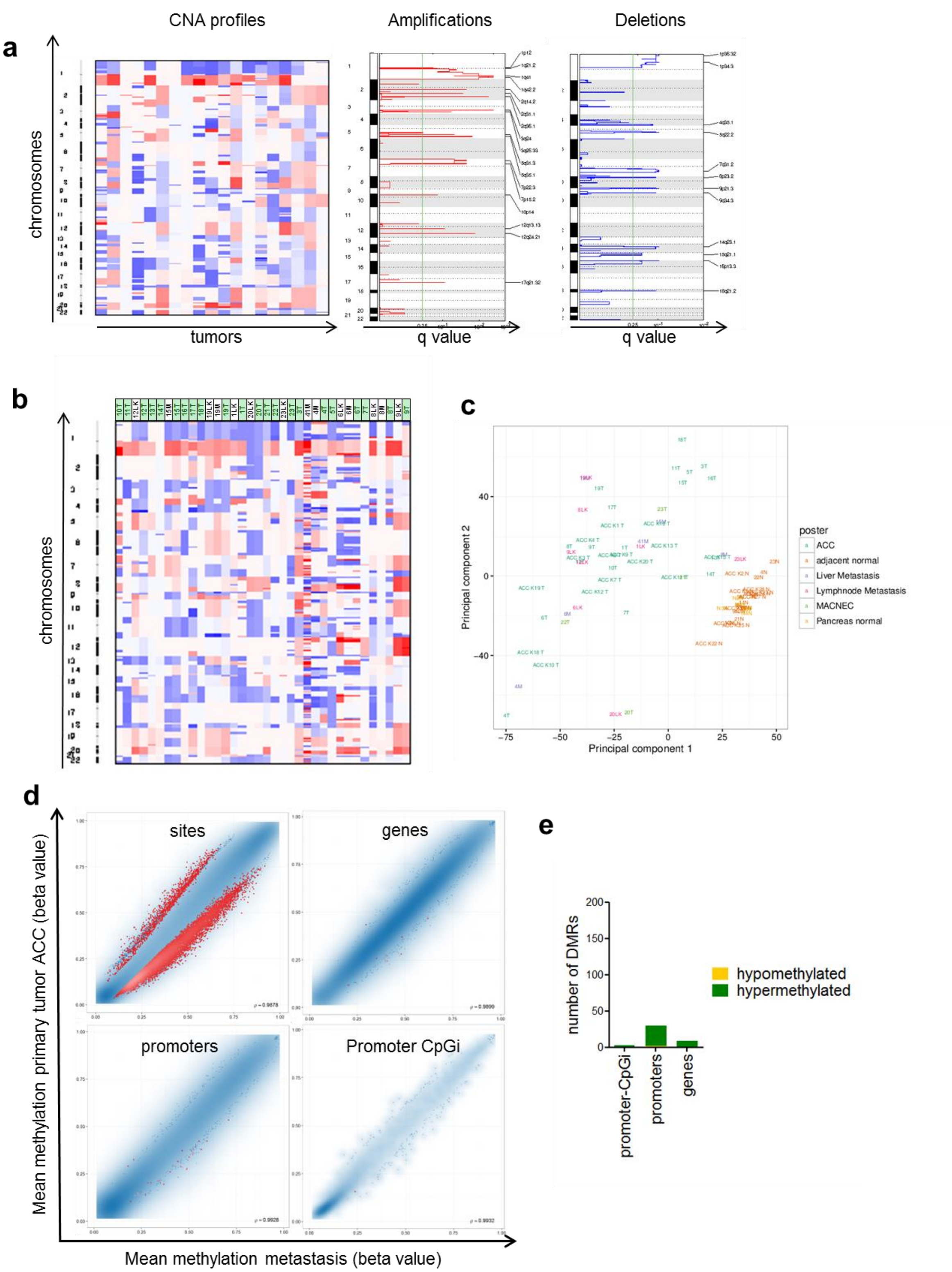

Supplementary Figure 3

ACC are characterized by numerous copy number changes, mainly deletions

a. CNA profiles: Heatmap of copy numbers calculated via the intensities of the 450K array (each tumor versus average normal) are displayed for each tumor of cohort 2 and each

chromosome (red: amplifications, blue: deletions). **Amplifications**: q-values of amplifications of all tumors of cohort 2. **Deletions**: q-values of deletions of all tumors of cohort 2. **b.** Heatmap of copy numbers displaying primary tumors and metastasis of cohort 1. (green: primary tumor, white: metastasis). **c.** PCA of all CpG sites of cohort 1 and cohort 2. Equal numbers correspond to one patient. Endings: N=normal, T=tumor, LK=lymph node metastasis, M=metastasis other than lymph nodes. **d.** Methylation scatterplot of all CpG sites, genes, promoters and promoter CpG islands (CpGi) in a paired analysis of metastasis versus primary tumors. (Red: sites are above the rank cutoff, e.g. are considered biologically significant, blue: transparency corresponds to density of sites). **e.** Number of differentially methylated promoter CpG islands, promoters and genes in a paired analysis of metastasis versus primary tumors.

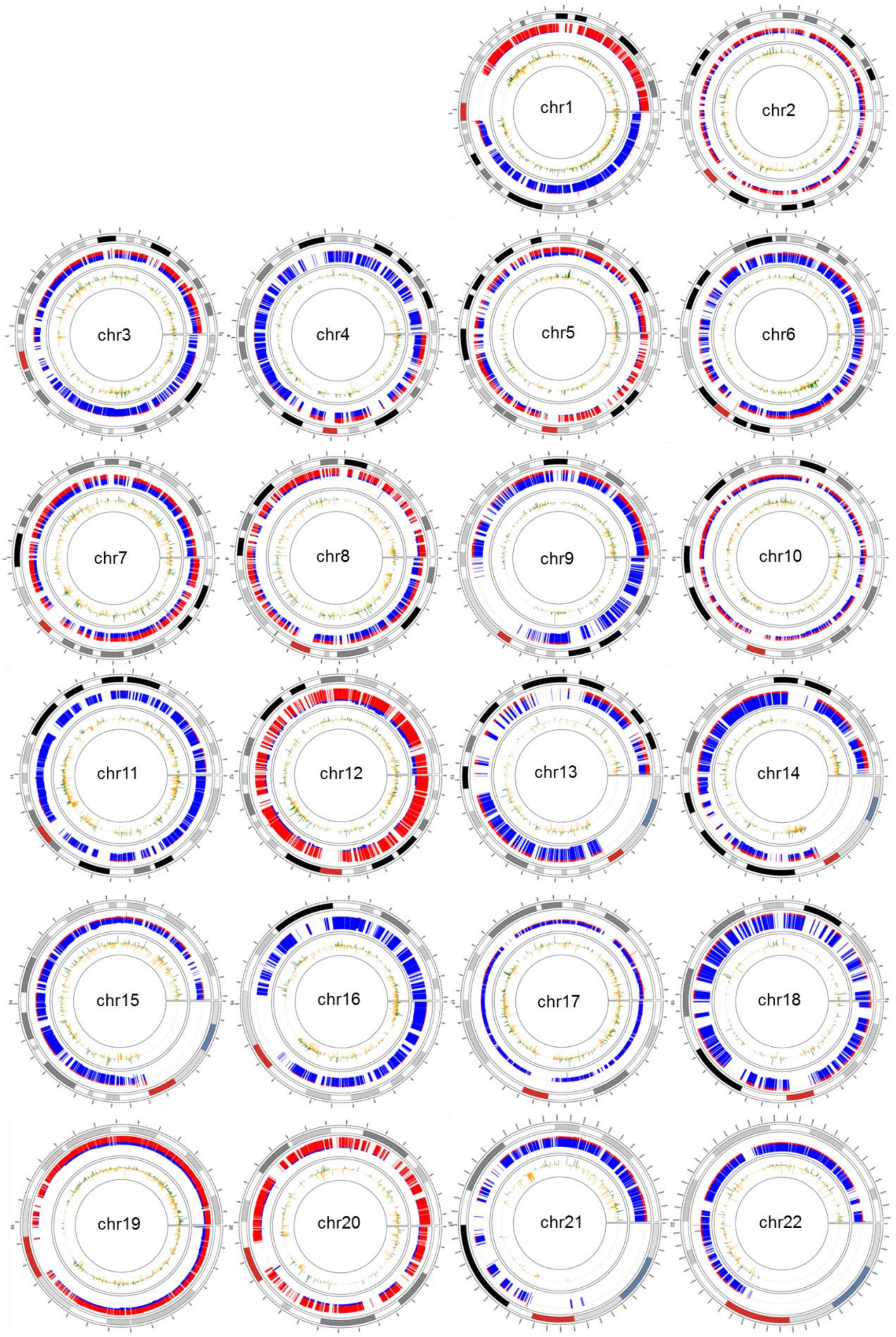
chr1
chr2
chr3
chr4
chr5
chr6
chr7
chr8
chr9
chr10
chr11
chr12
chr13
chr14
chr15
chr16
chr17
chr18
chr19
chr20
chr21
chr22

Supplementary Figure 4
Circus plot displaying each autosome (outer circle, grey, centrosomes red) with amplifications (middle circle, red), deletions (middle circle, blue), hypermethylation (inner circle, green), hypomethylation (inner circle, yellow).

52

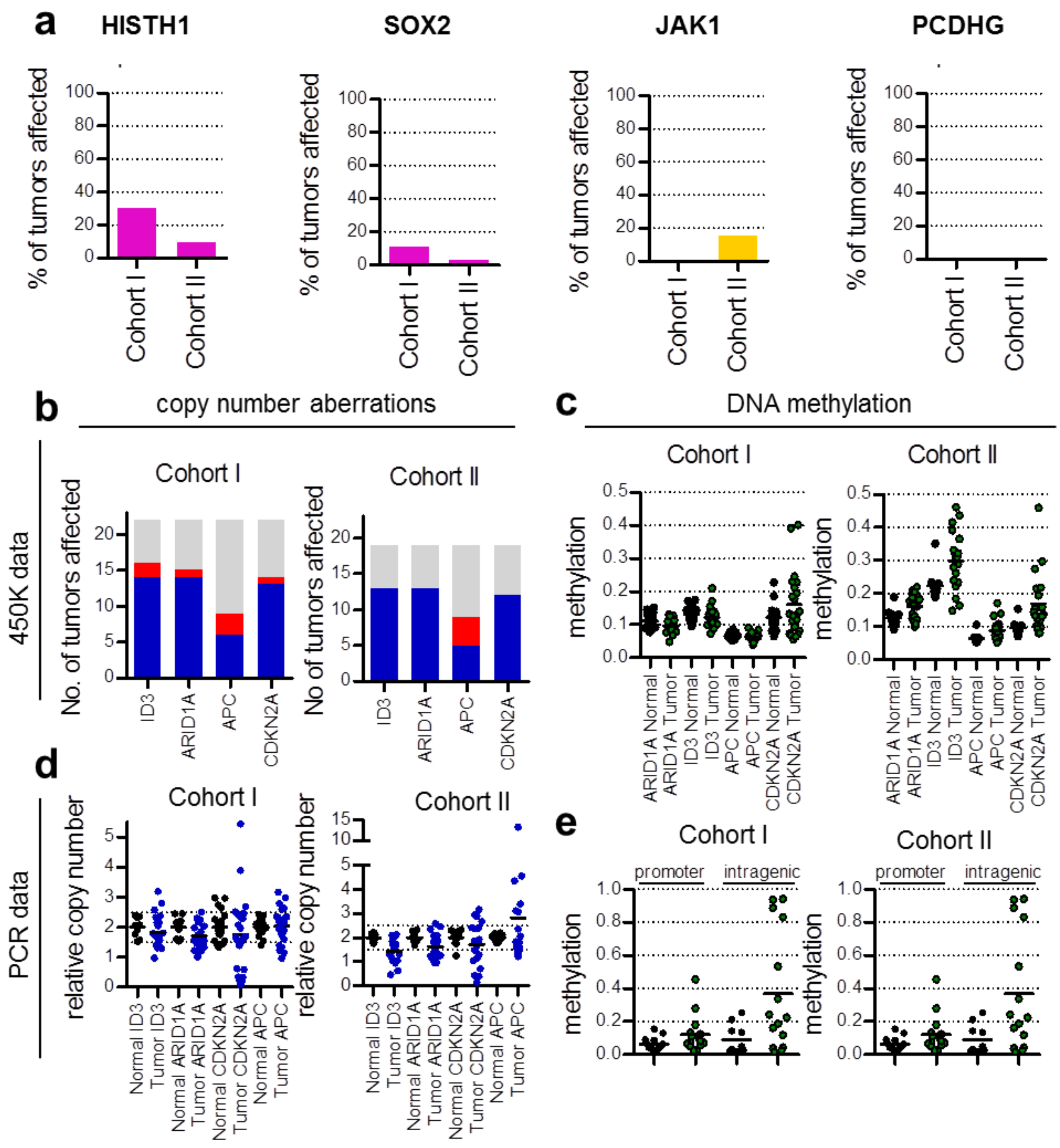

Supplementary Figure 5

Protein expression, copy number alterations and promoter methylation of cohort 1 and 2 of top candidates

a. Percentage of tumors revealing aberrations in protein expression of HISTH1 overexpression, SOX2 overexpression, JAK1 downregulation, and PCDHG downregulation determined by IHC

b-e. Data for ID3, ARID1A, APC, and CDKN2A; b. Number of tumors affected by deletion or

amplifications calculated via 450K data. **c.** Promoter methylation measured by 450K. Each dot represents one sample; black= normal tissue, green= tumor tissue. **d.** Copy number aberrations measured via qPCR. Each dot represents one sample; black= normal tissue, blue= tumor tissue. **e.** CDKN2A, promoter methylation and intragenic methylation measured by MassARRAY. Each dot represents one sample; black= normal tissue, green= tumor tissue.

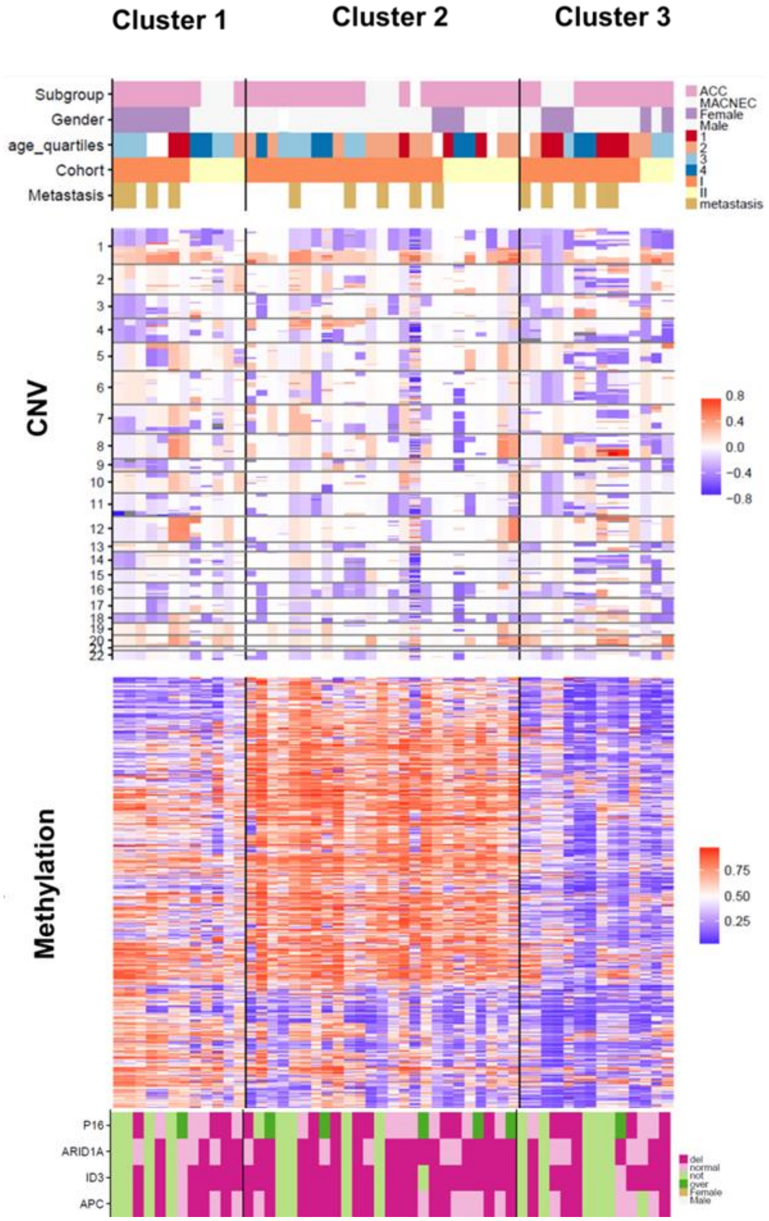

Supplementary Figure 6
Molecular subgroups of ACC can be identified with 450K data
a. Unsupervised clustering using the methylation data set and copy number aberrations from 450K reveal three different subgroups within the ACC tumors (both cohorts). The underlying cause of these clusters cannot be explained by the available clinical data (top panel) or molecular alterations (lower panel) and remains to be elucidated.

Supplementary Table 1
Patient characteristics and samples used for 450K analysis, WEES and IHC

	Cohort I	Cohort II
**Total number of tumors**	**34**	**39**
**General characteristics**		
Female:Male (%female)	6:17 (26.1%)	11:28 (28.2%)
Mean age in years (range)	56.5 (29-75)	58.2 (7-79)
median survival in months (interquartile range)	26 (16-121)	NA
**Mutational analysis - Exome sequencing (FFPE+FF)**		
Primary tumors(pure ACCs, MACNECs)	22 (18,4) (5FF+17FFPE)	1 FFPE
Normal tissues (adjacent healthy pancreas, healthy liver)	22 (19,3)	1
**Methylation analysis and Copy number aberrations - 450K Array (FFPE)**		
Primary tumors (pure ACCs, MACNECs)	22 (18, 4)	19 (15, 4)
Metastases	12 (6 lymph nodes , 5 liver, 1 peritoneal)	NA
Normal tissues (adjacent)	20 (14)	10 (2)
**Protein analysis - IHC (FFPE)**		
Primary tumors(pure ACCs, MACNECs)	20 (16,4)	38 (30,8)
Metastases	3 (liver)	1 (peritoneal)
Normal tissues	8	

Supplementary Methods

Estimation of tumor purity

Additionally to the high tumor purity assessment of the pathologist, tumor purity was estimated via the LUMP (leukocytes unmethylation for purity) method[1], which correlates well with the other tumor purity methods ESTIMATE and ABSOLUTE[1]. To that end, 44 immune-specific CpG site methylation levels were averaged and divided by 0.85[1].

MassARRAY analysis of DNA methylation in candidate regions

MassARRAY is a mass spectrometry based method which quantifies methylation levels of specific candidate regions[2]. DNA was bisulfite treated as described above. Regions of interest were amplified using conventional PCR with primers specific for bisulfite-converted DNA, followed by *in vitro* transcription and fragmentation in a base specific way and run on the MassARRAY (Agena Bioscience, San Diego, US) which separates fragments due to their mass to charge ratio. The shift coming from an unmethylated to a methylated fragment will result in quantitative methylation values. For primer sequences refer to Supplementary Data FileTable 11.

Quantitative PCR analysis of CNA in candidate regions

Copy numbers were evaluated by performing quantitative PCR on the target regions via the LightCycler 480 System (Roche, Basel, Switzerland). Actin β (ACTB) and Albumin (ALB) were used as housekeeping genes. Copy numbers were calculated as follows:

$$\mathrm{Y} = \mathrm{Eff_{GOI}}^{-\mathrm{C_{T(GOI)}}} - \mathrm{average}(\mathrm{Eff_{ACTB}}^{-\mathrm{C_{T(ACTB)}}} + \mathrm{Eff_{ALB}}^{-\mathrm{C_{T(ALB)}}})$$

$$\text{relative DNA content (Sample)} = \frac{\mathrm{Y_{Sample}}}{\mathrm{Y_{average\ normals}}} \times 2$$

For primer sequences refer to Supplementary Data File 11.

Molecular subgroups of ACC

iCluster Plus[3] was used to investigate molecular subgroups of ACC. Three Clusters were determined to explain the highest variation while showing the least complexity. Definition of these clusters was only based on copy number alterations and promoter DNA methylation, as point mutations did not add any information to these clusters.

Supplementary References

1. Aran, D., Sirota, M. & Butte, A.J. Systematic pan-cancer analysis of tumour purity. *Nat Commun* **6**, 8971 (2015).
2. Ehrich, M. *et al.* Quantitative high-throughput analysis of DNA methylation patterns by base-specific cleavage and mass spectrometry. *Proc Natl Acad Sci U S A* **102**, 15785-90 (2005).
3. Mo, Q. & Shen, R. iClusterPlus: Integrative clustering of multi-type genomic data. R package version 1.9.0. (2013).
