# Supplementary material for: Genome-wide genetic and epigenetic analyses of pancreatic acinar cell carcinomas reveal aberrations in genome stability
Source: Nat Commun. 2017 Nov 6;8:1323. doi: 10.1038/s41467-017-01118-x (PMC5673892; doi:10.1038/s41467-017-01118-x)
Supplement: Supplementary file 3 — Description of Additional Supplementary Files [file 41467_2017_1118_MOESM3_ESM.pdf]

Description of Additional Supplementary Files

File Name: Supplementary Data 1
Description: Detailed results from WES. 0/1: heterozygous point mutation, 1/1 homozygous point mutation and MSI status of a subset of tumors.

File Name: Supplementary Data 2
Description: Contribution of COSMIC signatures to mutational pattern in ACC.

File Name: Supplementary Data 3
Description: Beta values of promoters, promoter CpG islands, CpG islands and gene bodies of cohort 1 and cohort 2.

File Name: Supplementary Data 4
Description: Differentially methylated regions (of promoters, promoter CpG islands, CpG islands and gene bodies) defined by the rank cutoff in cohort 1.

File Name: Supplementary Data 5
Description: Differentially methylated regions (of promoters, promoter CpG islands, CpG islands and gene bodies) defined by the rank cutoff in cohort 2.

File Name: Supplementary Data 6
Description: Validated genes with aberrant methylation at gene bodies, promoters and promoter CpG islands.

File Name: Supplementary Data 7
Description: Deleted and amplified regions and the genes mapping to those regions in cohort 1 and cohort 2.

File Name: Supplementary Data 8
Description: Matrix of amplified and deleted genes, containing each gene and each tumor in cohort 1 and cohort2.

File Name: Supplementary Data 9
Description: Integrated list of hits in methylation and CNA overlapped with previously published lists (for details refer to methods section).

File Name: Supplementary Data 10
Description: Drugs for potential basket trials in ACC that exploit the identified molecular aberrations31,32 are already available either in clinical trials or as approved drugs for other cancer entities33,34.

File Name: Supplementary Data 11
Description: Primer sequences and antibodies used for MassARRAY, qPCR and IHC.
